# Magnetoelectric relaxor and reentrant behaviours in multiferroic Pb(Fe_2/3_W_1/3_)O_3_ crystal

**DOI:** 10.1038/srep22327

**Published:** 2016-03-03

**Authors:** Ling Chen, Alexei A. Bokov, Weimin Zhu, Hua Wu, Jian Zhuang, Nan Zhang, Hamel N. Tailor, Wei Ren, Zuo-Guang Ye

**Affiliations:** 1 Electronic Materials Research Laboratory, Key Laboratory of the Ministry of Education & International Center for Dielectric Research, Xi’an Jiaotong University, Xi’an 710049, China; 2 Department of Chemistry and 4D LABS, Simon Fraser University, Burnaby, British Columbia, V5A 1S6, Canada; 3 Department of Applied Physics, Donghua University, Songjiang District, 201620 Shanghai, P.R. China; 4 School of Mathematics and Physics, China University of Geosciences (Wuhan), Wuhan 430074, P.R. China

## Abstract

Significant quenched disorder in crystal structure can break ferroic (magnetic or electric) long-range order, resulting in the development of ferroic glassy states at low temperatures such as magnetic spin glasses, electric dipolar glasses, relaxor ferroelectrics, etc. These states have been widely studied due to novel physical phenomena they reveal. Much less known are the effects of quenched disorder in multiferroics, i.e. the materials where magnetic and electric correlations coexist. Here we report an unusual behaviour in complex perovskite Pb(Fe_2/3_W_1/3_)O_3_ (PFW) crystals: the coexistence of electric relaxor, magnetic relaxor and antiferromagnetic (AFM) states. The most striking finding is the transformation of the AFM phase into a new reentrant-type magnetic glassy phase below *T*_*g*_ ≅ 10 K. We show that the behaviour at this transformation contrasts the typical behaviour of canonical spin glasses and is similar to the behaviour of relaxor ferroelectrics. Magnetoelectric effect is also observed in the AFM phase in the temperature range of the transition into electric relaxor phase at *T*_*f*_ ≅ 200. The mechanism of magnetic relaxor behaviour is supposed to arise from the frustrated interactions among the spins located at the AFM domain walls. Our results should inspire further studies of multirelaxor behaviour in other multiferroic systems.

Multiferroic materials which exhibit both magnetic and ferroelectric orders and effects of coupling between magnetization and electric polarization have attracted much interest in the last decade and are considered to be extremely promising for a number of advanced applications such as spintronics and high-density data storage^1^,^2^,^3^. Of particular interest is the behaviour of the materials in which disordered structure is associated with the development of magnetic or electric glassy states. These states generally reveal some analogous features, such as splitting of field-cooling (FC) and zero-field-cooling (ZFC) magnetization (polarization), frequency-dependent maximum in the temperature dependence of magnetic (electric) ac susceptibility, etc. On the other hand, it remains unclear how far this analogy can be extended. In particular, relaxor ferroelectrics are the most widely studied electric glassy systems^4^,^5^, but the existence of relaxor ferromagnetic state has been reported only recently^6^.

Relaxor ferroelectrics are the crystalline materials whose structure is characterised by the presence of quenched (static) chemical disorder in the arrangement of different atoms on the equivalent crystallographic positions. Similar to normal ferroelectrics, the polar order appears in relaxors upon cooling at a particular temperature, but contrary to normal ferroelectrics, this order is local or short-ranged (i.e. develops only in nanoscale regions) and the dipolar arrangement inside the polar nanoregions is still a subject of intense debates. The dipolar transformation in relaxors takes place over a wide temperature range (typically hundreds of degrees) in which unusual properties (in particular, extraordinary dielectric relaxation) are observed.

Magnetoelectric correlations in disordered glassy ferroics have been poorly understood so far, mainly because only in a few of them the ferroelectric and ferromagnetic (FM) properties have actually been observed simultaneously. In perovskite solid solutions of Sr_0.98_Mn_0.02_TiO_3_, frustrated dipolar and magnetic superexchange interactions give rise to both dipolar glass and spin glass states^7^. A new class of superparamagnetic state was found in the relaxor ferroelectric perovskite solid solution of BiFeO_3_-BaTiO_3_, where magnetic nanodomains were thought to be induced by electric polar nanoregions and to overlap with them in space^8^. Coexistence of magnetic relaxor and electric relaxor states was reported in perovskite Pb(Fe_1/2_Nb_1/2_)O_3_-Pb(Mg_1/2_W_1/2_)O_3_ ceramics^6^. In this material significant magnetoelectric effect was observed at the electric relaxor freezing temperature. Ferroelectric, spin cluster glass and AFM states were found to coexist in PbFe_0.5_Nb_0.5_O_3_^9^.

In the present work we study the complex perovskite Pb(Fe_2/3_W_1/3_)O_3_ (PFW) crystals in which alongside the electric relaxor phase below *T*_*f*_ ≅ 200 K and the AFM phase with a Neel temperature, *T*_*N*_ = 350 K, we have found a new reentrant-type magnetic relaxor phase below *T*_*g*_ ≅ 10 K. The structure of PFW determined by neutron and x-ray diffraction methods is cubic at all temperatures with the space group *Pm-3m*, implying a fully disordered distribution of the Fe^3+^ and W^6+^ cations on the perovskite B-sites^10^,^11^. The G-type magnetic structure arising from the Fe^3+^-O-Fe^3+^ superexchange interactions was also found^10^ in PFW. The value of Néel temperature *T*_*N*_ = (340–360) K was estimated from the anomaly in the temperature dependence of magnetic susceptibility^10^,^12^. Another anomaly observed at about 10 K was attributed to the transition from the AFM phase to a weak FM state^13^. The dielectric characterization of PFW revealed a typical relaxor ferroelectric behaviour^14^.

## Results

**Figure 1:**
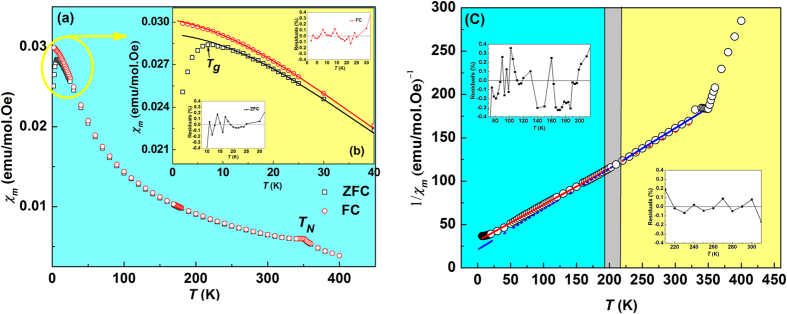
Magnetic properties of PFW crystal measured in a large dc field: (**a**) FC and ZFC magnetic susceptibilities vs *T*. (**b**) Magnification of the low-temperature part of (**a**). Solid lines are the fittings to quadratic equation (2). (**c**) Reciprocal FC susceptibility vs *T.* Solid lines are the fittings to the CW law (3) in the temperature intervals of 80 K ≤ *T* ≤ 190 K and 220 K ≤ *T* ≤ 300 K, respectively. Insets in (**b**,**c**) show the residual error analysis.

**Figure 2:**
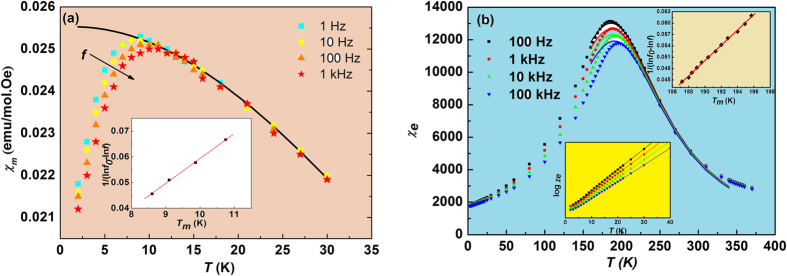
Comparison of electric and magnetic low-field properties of the PFW crystal. Temperature dependences of (**a**) magnetic and (**b**) electric ac susceptibility. Solid lines are fittings to equation (2) for *χ*_*e*_(*T*) at different frequencies and for *χ*_*m*_(*T*) at 1 Hz. Insets show the fitting to the Vogel-Fulcher law (1). Inset in (**b**) is the enlarged low-temperature part of the data plotted on logarithmic scale.

Figure 1(a) shows the temperature dependences of the ZFC and FC magnetic susceptibilities calculated according to *χ*_*m*_ = *M*/*H*. A cusp-like anomaly is observed at 350 K, which was also reported in earlier investigations and is related to the temperature *T*_*N*_^10^,^12^,^13^. The ZFC curve strongly deviates from the FC one below *T*_*g*_ ≈ 10 K, pointing to a nonergodic nature of the low-temperature state. To elucidate the origin of this nonergodicity the low-field ac *χ*_*m*_ was measured. The resulting temperature dependences are shown in Fig 2(a). A frequency dispersion at the low-temperature part of the diffuse *χ*_*m*_(*T*) peak is observed. The temperature of the maximum susceptibility (*T*_*m*_) varies with frequency, which excludes the possibility of FM or AFM transition as the origin of the *χ*_*m*_(*T*) peak: dispersion in conventional magnets can be observed only at much higher frequencies (mega to giga Hz). Therefore, a spin glass-type nature of the low-temperature state of PFW has to be considered. Since this glassy state is observed in the temperature range below the ordered AFM phase, it points to a reentrant behaviour. However, we cannot conclude if the AFM order is destroyed and thereby a pure glassy phase is formed, or the glassy and AFM orders coexist at low temperatures (neutron diffraction experiments which could answer this question unambiguously were performed in PFW only down to 10 K^10^,^15^).

Figure 2(b) presents the variation of ac electric susceptibility as a function of temperature measured at various frequencies. One can see an obvious similarity to the magnetic behaviour. Furthermore, we will show that the temperature and frequency variations of *χ*_*m*_ and *χ*_*e*_ in PFW can be described by the same empirical relations. The observed susceptibility behaviour is characteristic of relaxor ferroelectrics and differs considerably from the behaviour of canonical spin glasses.

In electric relaxors^5^,^16^ the frequency ( *f* ) shift of *T*_*m*_ is known to follow the Vogel-Fulcher (VF) law:


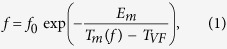


and the shape of the high-temperature slope of the *χ*(*T*) peak is described by a Lorentzian-type function:


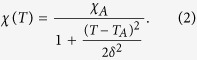


In the above equations *χ*_*A*_ and *T*_*A*_ (<*T*_*m*_) are the magnitude and the (maximum) temperature of the Lorentzian peak, respectively, *δ* is the half-width of the peak at 2/3 of the maximum, and *E*_*m*_ and *T*_*VF*_ are the parameters. If the high-temperature slope of the peak is frequency independent (as observed in many practical cases), the measured *χ* represents the static value of susceptibility. It was shown^16^,^17^ that low-field static susceptibility in electric relaxors follows equation (2) not only above *T*_*A*_, but also at and below *T*_*A*_, where it cannot be directly measured.

The VF law (1) is known to be also valid for *χ*_*m*_ in spin glasses^18^, but fulfilment of equation (2) has not been reported for magnets so far. Instead of diffuse maximum, the low-field dc susceptibility in canonical spin glasses shows a sharp cusp-like peak in the ZFC regime or a clear kink in the plateau in the FC regime. The sharp *χ*_*m*_(*T*) peak which is observed also in low-field ac measurements^19^,^20^ is believed to be the “hallmark” of spin glass behaviour^21^. The susceptibility typically reveals the Curie-Weiss (CW) temperature dependence upon cooling in the paramagnetic phase until the temperature is just above *T*_*m*_^20^,^22^. However, if the field strength increases, the cusp becomes rounded and deviation from the CW law is observed in a wide temperature interval between *T*_*m*_ and 2*T*_*m*_^19^,^23^,^24^,^25^.

Similar to other relaxor ferroelectrics, the *T*_*m*_(* f* ) dependence in PFW can be reasonably well fitted to the VF law [see inset in Fig. 2(b) and Table 1] with the best-fit parameters close to those observed in classical relaxors^17^,^26^. The fulfilment of equation (2) is also evident from Fig. 2b. The high-temperature slope of the *χ*_*e*_(*T*) peak is slightly *f*-dependent due to contribution from mobile charge carriers (the so-called universal relaxor dispersion often observed in relaxors, see ref. 16). The parameters of equation (2) presented in Table 1 were obtained at 100 kHz, where the universal relaxor contribution is negligible.

The characteristic relaxor equations (1) and (2) also hold for low-field ac magnetic susceptibility, as is evident from the fitting results presented in Table 1 and in Fig. 2a. The fitting parameters of equation (2) for *χ*_*m*_(*T*) curves obtained at different frequencies are found to be the same, which is consistent with the static nature of susceptibility above *T*_*m*_. The deviation of measured data from the fitted curve observed below the temperature where the dispersion begins (10–12 K) indicates that the measured susceptibility is not static. We also repeated fitting with modified equation (2) containing additional adjustable parameter, namely (*χ*_*A*_/*χ*_*m*_−1) ∝ (*T*−*T*_*A*_)^*γ*^ and obtained the value *γ* = 2.03 ± 0.09, which further confirms that equation (2) is robustly valid.

To verify the behaviour at temperatures around and below *T*_*m*_, we fitted to equation (2) our FC *χ*_*m*_(*T*) data which also represent static susceptibility, keeping in mind that they were obtained at a large field *H* = 200 Oe. As shown in Fig 1(b), in this case equation (2) appears to be valid at all temperatures below about 40 K. For the ZFC susceptibility (*H* = 100 Oe), deviation is observed at temperature lower than 10 K [see Fig. 1(b)], which is evidently because the measured *χ*_*m*_ has non-equilibrium values here. The best-fit parameters *T*_*A*_ and *δ* are practically the same for both the ZFC and FC regimes (see Table 1), which is expected, as the fitting was performed in the temperature range of 10–40 K, where equilibrium values of *χ*_*m*_ are reached. On the other hand, comparison with the ac *χ*_*m*_ reveals a significantly lower dc value of *T*_*A*_ (Table 1), which can be attributed to a higher measurement field. However, the difference in the values of diffuseness parameter *δ* remains insignificant. This behaviour contrasts with that of classical spin glasses, in which, as discussed above, the shape of the *χ*_*m*_(*T*) peak depends on the measurement field dramatically^19^,^24^,^25^.

While the quadratic law (2) is observed in relaxors in the vicinity of *T*_*m*_, above a much higher temperature *T*_*B*_ (*T*_*B*_ −*T*_*m*_ > *δ*), the susceptibility typically follows the CW law


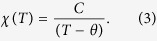


Figure 1(c) shows the CW fitting for the FC magnetic susceptibility in PFW (for ZFC similar results were obtained). The CW law indeed holds in a wide temperature range at *T* ≫ *T*_*m*_. Furthermore, the parameters of the law vary from *θ* = −75.5 K, *C* = 2.47 emu K/mol·Oe in the range of (80–190) K to *θ* = −42 K, *C* = 2.18 emu K/mol·Oe in the range of (220–300) K. The intermediate temperature interval of (190–220) K corresponds to the *χ*_*e*_(*T*) maximum, suggesting that the magnetic anomaly at these temperatures is caused by magnetoelectric coupling. On the other hand, no dielectric anomalies were observed in the temperature range of *χ*_*m*_(*T*) maximum: the *χ*_*e*_(*T*) dependences were found to be strictly linear in semilogarithmic scale (see inset in Fig. 2b).

**Figure 3:**
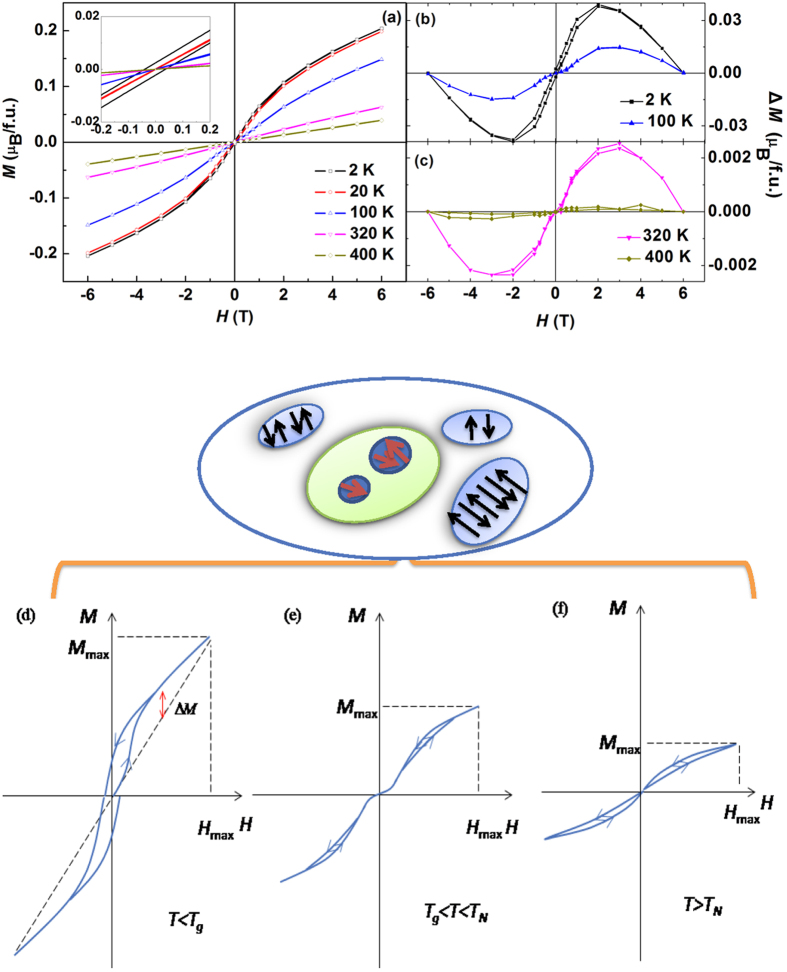
Magnetic behavior of PFW crystal at different temperatures: (**a**) Magnetic hysteresis loops after ZFC. Inset is a magnified low-field part of the curves; (**b**,**c**) Values of ΔM(*H*) loops calculated from hysteresis loops shown in (**a**); (**d**–**f**) Schematics of loops displayed in different temperature intervals. Line curvature and hysteresis are exaggerated to highlight the features.

Figure 3(a) shows the *M*(*H*) curves. Below *T*_*g*_ significant non-linearity and slim hysteresis loops with small remanent magnetization are observed, confirming the nonergodic nature of the low-temperature phase. The initial magnetization curve reveals an S-shaped form characteristic of both canonical and nonmetallic spin glasses^27^. With increasing temperature, the non-linearity decreases, but does not disappear even at *T* > *T*_*N.*_ Furthermore, while remanent magnetization is absent at *T* > *T*_*g*_, the irreversibility remains. The increasing and decreasing branches of *M*(*H*) loop do not coincide at large *H*, giving rise to a rather unusual double hysteresis loop. The S-shaped behaviour is conserved at *T* > *T*_*g*_ and disappears at *T* > *T*_*N*_. We calculated the deviation of the measured *M*(*H*) from the linear trend, 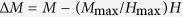, where *H*_*max*_ (=6 T) and *M*_*max*_(*T*) are the maximum measurement field and maximum obtained magnetization, respectively. Figure 3(b,c) show the Δ*M*(*H*) loops at selected temperatures and in Fig. 3(d–f) the *M*(*H*) loops are represented schematically.

## Discussion

To the best of our knowledge, double hysteresis loops were found in magnetic materials in two cases where they were the result of large uniaxial anisotropy. In AFM crystals with very strong uniaxial magnetocrystalline anisotropy irreversibility appears due to field-induced transition from AFM to FM order (metamagnetic transition)^28^,^29^,^30^. In some CuMn alloys where spin glass and FM orders coexist, anisotropy originates from the molecular field of FM domains^31^. However, uniaxial anisotropy is not consistent with cubic symmetry of PFW in the paramagnetic phase, and FM domains in PFW are absent.

Our explanation of the unusual magnetic behaviour of PFW implies the existence of local antiferromagnetically ordered regions surrounded by paramagnetic matrix in a significant temperature range above *T*_*N*_. Such a kind of non-percolating AFM clusters (AFMCs) is expected due to the same reasons as those proposed to justify the Griffiths phase in randomly diluted ferromagnets and antiferromagnets^32^,^33^. Ordered clusters in the Griffiths phase are usually supposed to be dynamic, but their size distribution and, accordingly, relaxation time distribution are very broad^34^. Consequently, some clusters are expected to be static on a practical time scale. Note that in contrast to FM transitions where the clusters of the low-temperature phase above *T*_*C*_ induce large demagnetizing field and, therefore, can be unstable, AFM clusters can easily be static, as they possess zero or small uncompensated magnetic moment producing no significant field.

A number of experimental facts reported in the literature support our idea about the existence of static AFM order at *T* > *T*_*N*_ in PFW. In particular, the spontaneous magnetization of AFM sublattices determined from the intensities of neutron diffraction magnetic peaks at *T*_*N*_ = 340 K amounts to ~40 % of the magnetization at *T* ~ 0 K and vanishes only at *T* ≅ 400 K (see Fig. 7 in Ref. 10). Magnetic ordering below 415 K was revealed in Mössbauer spectra^35^. The CW law with *θ* < 0 K (as expected for paramagnetic phase of antiferromagnets) is observed only above ~450 K^12^.

**Figure 4:**
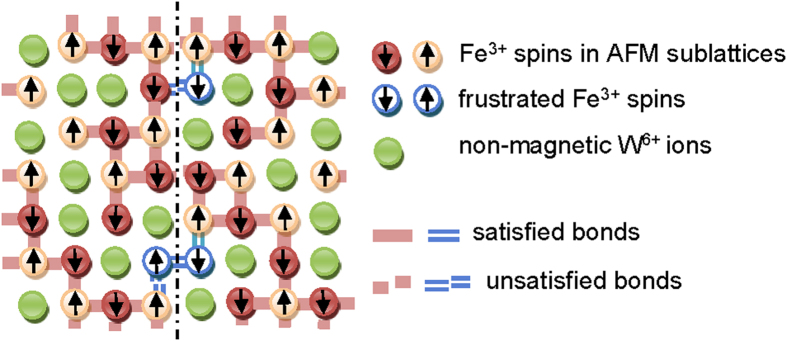
Schematic representation of two AFM domains in PFW. Domain boundary is shown by dashed line. Due to the existence of competing bonds (shown blue) some spins (blue) appear to be frustrated.

The infinite AFM cluster seems to appear upon cooling at *T*_*N*_ as a result of growth and merging of randomly arranged AFMCs. As the AFMCs were static before merging, the directions of their AFM sublattices are conserved in the course of the phase transition and the infinite AFM cluster appears to be divided into a large number of small domains. Figure 4 illustrates the domain structure in the diluted AFE phase of PFW. As the bonds are unsatisfied along the domain wall, it is characterized by enhanced energy. In a diluted AFE crystal the walls tend to be located in the regions rich in non-magnetic ions in order to reduce the number of unsatisfied bonds and, thereby, the domain wall energy. Some spins adjacent to the wall and surrounded by a large number of non-magnetic ions may appear in the positions where they are subject to frustrated interactions, as shown schematically in Fig. 4. These can be single spins (as shown on the top part of Fig. 4) as well as clusters of two or more spins (bottom part of Fig. 4.) These spins have a comparatively small number of bonds, thus the energy barriers for their reorientation are small and the spins remain dynamically disordered in the AFE phase, contributing to the CW behaviour. The irreversibility and relaxation phenomena observed at *T* < *T*_*g*_ ≈ 10 K can be related to glassy freezing of these dynamic spins.

Figure 4 represents a simple model of Ising antiferromagnet where two directions of spins are possible (up or down). In real PFW crystal various spin directions are allowed, which should give rise to comparatively thick (Bloch-like or Néel-like) domain walls of various orientations^36^. Within a wall, the reorientation of a frustrated spin can influence the orientations of many faraway spins. In this way coupling between different frustrated spins can be presumably achieved and the global coherence of glassy state can appear.

The coexistence of unsaturated bonds with static AFMCs and AFM domains at *T* > *T*_*N*_ and *T* < *T*_*N*_, respectively, can also explain the origin of observed double hysteresis loops. Static AFM order creates uniaxial anisotropy, which, as discussed above, is essential for the development of double hysteresis loops. In the ZFC process, the uncompensated moments of AFMCs are randomly oriented. An external magnetic field cannot reorient the whole magnetic moment of AFM sublattice inside AFMCs (the reorientation would not give rise to a decrease in energy), however, the moments of those Fe^3+^ ions belonging to AFMCs whose bonds are not saturated (especially at the boundaries of AFMCs) can be reoriented in a large enough field and appear in metastable states. These reorientations lead to the observed *M*(*H*) nonlinearity and double hysteresis loops. When the external field is removed, individual moments switch back to the stable state so that the remanent magnetization is absent.

Our observation of the AFM phase transition and the magnetic and electric relaxor behaviour entitles PFW to be classified as a multirelaxor with reentrant magnetic freezing. The single compound (as opposed to other multirelaxors found in the form of solid solutions) and the diversity of phase sequence make this material a model system for studying multiferroic phenomena in disordered materials.

## Methods

The PFW crystals which we studied were grown from high temperature solution^14^. Magnetization (*M*) of the crystal oriented along the [001] crystallographic direction was determined with a SQUID magnetometer (Quantum Design MPMS XL). Before each measurement the sample was heated up to 400 K without magnetic field. Magnetic moment was measured upon cooling with a field of *H* = 200 Oe (in FC experiments) or the sample was cooled down to 2 K without the field, and afterwards the moment was measured upon heating with *H* = 100 Oe (in ZFC experiments). The isothermal *M*(*H*) dependences were measured at different temperatures after cooling in zero field from 400 K. The ac magnetic susceptibility (*χ*_*m*_) was measured under the ZFC mode with *H* = 5 Oe. Electric ac susceptibility (*χ*_*e*_) was studied using a Novocontrol Alpha broadband dielectric spectrometer. The measured dependences were fitted to empirical formulae using nonlinear least squares method.

## Additional Information

**How to cite this article**: Chen, L. *et al.* Magnetoelectric relaxor and reentrant behaviours in multiferroic Pb(Fe_2/3_W_1/3_)O_3_ crystal. *Sci. Rep.* **6**, 22327; doi: 10.1038/srep22327 (2016).

## Figures and Tables

**Table 1:** The best-fit parameters of equations (1) and (2) for electric, *χ*_*e*_, and magnetic, *χ*_*m*_, susceptibilities.

	*χ*_*e*_	*χ*_*m*_		
		Low-field ac	FC dc	ZFC dc
*T*_*VF*_ (K)	164	5.4		
*E*_*m*_ (K)	560	80.2		
*f*_0_ (Hz)	2 × 10^12^	3.2 × 10^9^		
*T*_*A*_ (K)	186	3.2 ± 0.7	−4.4 ± 0.4	−4.0 ± 0.5
*δ* (*K*)	59	45.5 ± 1.1	53.4 ± 1.2	55.0 ± 0.7

## References

[b1] Bibes M. Nanoferronics is a winning combination. Nature Mater. 11, 354–357 (2012). 22522625 10.1038/nmat3318

[b2] Tokura Y. Multiferroics as Quantum Electromagnets. Science 312, 1481–1482 (2006). 16763137 10.1126/science.1125227

[b3] Kleemann W., Borisov P., Bedanta S. & Shvartsman V. V. Multiferroic and Magnetoelectric Materials Novel Developments and Perspectives. IEEE Trans. Ultrason. Ferroel. Freq. Control 57, 2228–2232 (2010). 10.1109/TUFFC.2010.1682 20889409

[b4] Cross L. E. Relaxor ferroelectrics. Ferroelectrics 76, 241–267 (1987).

[b5] Bokov A. A. & Ye Z.-G. Recent progress in relaxor ferroelectrics with perovskite structure. J. Mater. Sci. 41, 31–52 (2006).

[b6] Levstik A. *et al.* Magnetoelectric relaxor. Appl. Phys. Lett. 91, 012905 (2007).

[b7] Shvartsman V. V. *et al.* (Sr;Mn)TiO_3_: A Magnetoelectric Multiglass. Phys. Rev. Let. 101, 165704 (2008). 18999688 10.1103/PhysRevLett.101.165704

[b8] Soda M., Matsuura M., Wakabayashi Y. & Hirota K. Superparamagnetism Induced by Polar Nanoregions in Relaxor Ferroelectric (1 - x)BiFeO_3_–xBaTiO_3_. J. Phys. Soc. Jpn. 80, 043705 (2011).

[b9] Kleemann W., Shvartsman V. V. & Borisov P. Coexistence of Antiferromagnetic and Spin Cluster Glass Order in the Magnetoelectric Relaxor Multiferroic PbFe_0.5_Nb_0.5_O_3_. Phys. Rev. Lett. 105, 257202 (2010). 21231620 10.1103/PhysRevLett.105.257202

[b10] Ivanov S. A., Eriksson S.-G., Tellgren R. & Rundlöf H. Neutron powder diffraction study of the magnetoelectric relaxor Pb(Fe_2/3_W_1/3_)O_3_. Mater. Res. Bull. 39, 2317–2328 (2004).

[b11] Mitoseriu L., Carnasciali M. M., Piaggio P. & Nanni P. Evidence of the relaxor-paraelectric phase transition in Pb(Fe_2/3_W_1/3_)O_3_ ceramics. Appl. Phys. Lett. 81, 5006 (2002).

[b12] Bokov V. A., Mylnikova I. E. & Smolenskii G. A. Ferroelectric antiferromagnetics. Zh. Eksp. Teor. Fiz. 42 643–645 (1962); *Sov. Phys. JETP* (English Transl.) 15 447-449 (1962).

[b13] Ye Z.-G., Toda K., Sato M., Kita E. & Schmid H. Synthesis, structure and properties of the magnetic relaxor ferroelectric Pb(Fe_2/3_W_1/3_)O_3_ [PFW]. J. Korean Phys. Soc. 32, S1028–S1030 (1998).

[b14] Ye Z.-G. & Schmid H. Growth from high temperature solution and characterization of Pb(Fe_2/3_W_1/3_)O_3_ single crystals. J. Cryst. Growth 167, 628–637 (1996).

[b15] Wong P.-Z. *et al.* A. Coexistence of spin-glass and antiferromagnetic orders in the Ising system Fe_0.55_Mg_0.45_Cl_2_. Phys. Rev. Lett. 55, 2043–2046 (1985). 10031995 10.1103/PhysRevLett.55.2043

[b16] Bokov A. A. & Ye Z.-G. Dielectric relaxation in relaxor ferroelectrics. J. Advanced Dielectrics 2, 1241010 (2012).

[b17] Bokov A. A. & Ye Z.-G. Double freezing of dielectric response in relaxor Pb(Mg_1/3_Nb_2/3_)O_3_ crystals. Phys. Rev. B 74, 132102 (2006).

[b18] Tholence J. L. On the frequency dependence of the transition temperature in spin glasses. Solid State Commun. 35, 113–117 (1980).

[b19] Cannella V. & Mydosh J. A. Magnetic ordering in gold-iron alloys. Phys Rev. B 6, 4220–4224 (1972).

[b20] Mulder C. A. M., van Duyneveldt A. J. & Mydosh J. A. Susceptibility of the C*u*Mg spin glass – frequency and field dependences. Phys. Rev. B 5723, 1384–1396 (1981).

[b21] Binder K. & Kob W. Glassy Materials and Disordered Solids (World Scientific, 2011) p. 234.

[b22] Nagata S., Keesom P. H. & Harrison H. R. Low-dc-field susceptibility of CuMn spin glass. Phys. Rev. B 19, 1633–1638 (1979)

[b23] Morgownik A. F. J. & Mydosh J. A. High-temperature susceptibility of the CuMn spin-glass. Phys Rev. B 24, 5277–5283 (1981).

[b24] Monod P. & Bouchiat H. Equilibrium magnetization of a spin-glass – is mean-field theory valid. Journal de Physique Lett. 43, L45–L53, (1982).

[b25] Chamberlin R. V., Hardiman M., Turkevich L. A. & Orbach R. Phys Rev. B 25, 6720 (1982).

[b26] Glazounov A. E. & Tagantsev A. K. Appl. Phys. Lett. 73, 856 (1998).

[b27] Binder K. & Young A. P. Spin glasses. Rev. Mod. Phys. 58, 801–976 (1986).

[b28] Buschow K. H. J. & De Boer F. R. Physics of magnetism and magnetic materials (Kluver Academic/Plenum Publishers, NY, 2003) p. 33.

[b29] de Groot C. H., Buschow K. H. J. & de Boer F. R. Magnetic properties of R_6_Fe_13−x_M_1+x_ compounds and their hydrides. Phys. Rev. B 57, 11472–11482 (1998).

[b30] Barandiaran J. M., Gutierrez J., Rodriguez Fernandez J., Amboage M. & Righi L. Anomalous hysteresis and metamagnetism in Bi substituted perovskites. Physica B 343, 379–383 (2004).

[b31] Senoussi S. Anisotropy in the zero-field-cooled states of Ni_79_Mn_21_ and Au_81_Fe_19_: Irreversibility effects. Phys. Rev. Lett. 51, 2218–2220 (1983).

[b32] Griffiths R. B. Nonanalytic behavior above the critical point in a random Ising ferromagnet. Phys. Rev. Lett. 23, 17–19 (1969).

[b33] Bray A. J. Nature of the Griffiths phase. Phys. Rev. Lett. 59, 586–589 (1987). 10035812 10.1103/PhysRevLett.59.586

[b34] Bray A. J. Dynamics of dilute magnets above *T*_*C*_. Phys. Rev. Lett. 60, 720–722 (1988). 10038629 10.1103/PhysRevLett.60.720

[b35] Venevtsev Y. N. *et al.* Investigation of system PbFe_2/3_W_1/3_O_3_ –BaTiO_3_. Kristallografiya 21, 971–975 (1976).

[b36] Bode M. *et al.* Atomic spin structure of antiferromagnetic domain walls. Nature Mater. 5, 477–481 (2006). 16680147 10.1038/nmat1646

